# Evaluation of the Effect of Commiphora wightii and Asthiposhak in Experimental Rat Model of Osteoarthritis

**DOI:** 10.7759/cureus.92597

**Published:** 2025-09-17

**Authors:** Achal A Sukhdeve, Yashashri C Shetty, Mukesh Chawda, Megha Nalawade, Minakshi Dongre

**Affiliations:** 1 Pharmacology and Therapeutics, Seth G S Medical College and KEM Hospital, Mumbai, IND; 2 Medical Services, Solumiks Herbaceuticals Limited, Mumbai, IND; 3 Clinical Research Associate, Shree Dhootapapeshwar Limited, Mumbai, IND

**Keywords:** antiarthritic, cartilage oligomeric matrix protein (comp), chondroprotective, grip strength test, matrix metalloproteinase-13 (mmp-13)

## Abstract

Background: In Western medicine, a lot of research is ongoing to find molecules for osteoarthritis (OA). Ayurvedic physicians have tried *Commiphora wightii* (CW) and Asthiposhak Tablets (ASTP) in the management of OA, which has not been investigated in animal models of OA. Our objectives were to evaluate the effect of *C. wightii* and Asthiposhak in the improvement of behavioural tests, histopathological scores, Cartilage Oligomeric Matrix Protein (COMP)/MMP-13 biomarkers by ELISA in animal models of OA.

Methodology: After (Institutional Animal Ethics Committee (IAEC)/GSMC/02/2023) animal ethics approval, seven groups (n=6, each group): Normal Control, Disease Control, Positive Control, *C. wightii* low dose (CW-LD), *C. wightii* high dose (CW-HD), Asthiposhak low dose (ASTP-LD), Asthiposhak high dose (ASTP-HD) groups were used to study the chondroprotective effect in monosodium-iodoacetic acid-induced osteoarthritis in rats. Behavioural assessments (Rotarod, Hot Plate, Grip Strength, and Open Field Tests) were done weekly. On day 28, COMP and Matrix Metalloproteinase-13 (MMP-13) in serum were estimated by ELISA. On day 29, samples were collected for knee joint histopathology. Data was analysed using appropriate statistical tests.

Results: In the behavioural test, rotarod and hot plate analgesiometer, it was found that CW, both doses, and ASTP-HD have shown good improvement in comparison to disease control. While in the grip strength and open field test, CW, both doses have shown significant improvement. In histopathology, CW*-LD* and CW-HD group scores were significantly reduced. MMP-13 and COMP levels were significantly decreased in both the *C. wightii* group as compared to the disease control.

Conclusion: CW both doses and ASTP high dose exhibited antiarthritic effect by virtue of its effects on behavioural tests, histology scores, and chondroprotective biomarkers.

## Introduction

Osteoarthritis (OA) is a degenerative joint disorder that is progressive and disabling [1]. It is a chronic condition affecting the entire joint, including the meniscus, ligament, articular cartilage, and periarticular muscle, and it can be caused by a variety of pathophysiological mechanisms [2]. The global prevalence of knee OA is estimated to be 16% and the incidence 203 per 10,000 person-years [3]. The prevalence and incidence ratios for females versus males are 1.69 and 1.39, respectively, and knee OA accounts for almost 80% of the OA burden worldwide [3]. The age-standardised prevalence of OA in India increased from 4,895 in 1990 to 5,313 in 2019 per 100,000 persons [4]. Similarly, disability-adjusted life years due to OA increased from 0.79 million to 2.12 million [4].

It has been suggested that inflammatory mediators such as cytokines (IL-1β), chemokines, and reactive oxygen species have a primarily destructive effect on articular cartilage. These inflammatory mediators lead to increased synthesis and release of matrix metalloproteinases (MMPs) and cartilage degradation [5]. The conservative approach to treatment includes aerobic exercise, local muscle strengthening, weight loss, education, shock-absorbing footwear, and oral analgesics such as acetaminophen and non-steroidal anti-inflammatory drugs. Invasive approaches include surgery such as arthroplasty, intra-articular corticosteroid injections, and hyaluronan injection [6]. However, even the most effective of these agents only yield clinically meaningful efficacy in half of those taking the drug, and the side effects and potential toxicities limit their use in a population that often has associated comorbidities. There is, therefore, a significant unmet need for new pharmacological treatments for OA [7].

Monosodium iodoacetate (MIA) is a metabolic inhibitor that indirectly destroys articular cartilage by inhibition of glyceraldehyde-3-phosphate dehydrogenase in chondrocytes. Glyceraldehyde-3-phosphate dehydrogenase plays a key role in the process of glycolysis, which supports chondrocyte nutrition. Depriving chondrocytes of their nutrition ultimately leads to their apoptosis and death. The MIA model was chosen by us in this study for its rapid induction of OA within 7 days and its ability to replicate cartilage lesions and functional joint impairment similar to those in humans. The induced OA resembles human degenerative OA in histological and pain-related behaviours [8].

Meloxicam is an oxicam derivative recommended by the US Food and Drug Administration for pain and inflammation management in OA. It is a selective COX-2 inhibitor and is commercially available in oral dosage form. In our study, we used meloxicam as the standard control drug, as it has been used as a positive control in most previous studies. Meloxicam was effective in improving the depth (cartilage degeneration score) and extension (total cartilage degeneration width) of the lesions in the MIA-induced OA. Moreover, at high doses, meloxicam (1 mg/kg) has a chondroprotective effect and shows protection against subchondral bone lesions and the deep cartilage. This functional unit might be essential for down-regulating persistent inflammation and the long-term degeneration pathway. The late-phase inflammatory process is alleviated by meloxicam treatment [9].

As there is no curative treatment for OA, and the treatment that is given has a lot of side effects associated with it, it remains a disease with unmet need. Ayurvedic herbs and formulations are widely used in the management of OA, such as the herb *Commiphora wightii* (CW) and formulations containing CW, one of which is Asthiposhak. CW possesses anti-inflammatory and analgesic properties and is preferred due to its potential to offer safe symptomatic relief and to protect the articular cartilage. CW is a small tree containing gum resin that is revered in Ayurveda for its medicinal properties. The plant’s oleoresin is known as gum guggul, containing 2% of Guggulsterones, a resin of the CW tree. The anti-inflammatory activity of CW has been attributed to its principal phytoconstituents, the guggulsterones. It is commonly referred to as gum guggul (oleoresin), which is used in the management of various musculoskeletal, metabolic, and skin disorders [10]. Asthiposhak is also reported for its bone-strengthening and anti-resorptive effects in participants suffering from osteopenia. Asthiposhak tablets exert beneficial effects on bone health due to the combined actions of their ingredients. A clinical study also indicated its potential in the management of joint disorders [11]. The existing medications for OA exert various distressing side effects and potential toxicities. Therefore, there is an unmet need for new pharmacological treatments in OA. Previous studies on CW have shown its efficacy on bone health in OA, but did not assess the biomarkers that are indicative of chondroprotection or cartilage damage. Hence, the current study was planned to include biomarkers along with additional behavioural tests to confirm the effect of CW in the OA rat model.

## Materials and methods

Before commencement of the study, permission was obtained from the Institutional Animal Ethics Committee (IAEC approval no: IAEC/GSMC/02/2023), which is registered with the Committee for Control and Supervision of Experiments on Animals (CCSEA). The animals used in this study were bred in the Central Animal House (60/PO/ReBi/S/1999/CPCSEA) of our institute. The study was conducted in accordance with the CCSEA guidelines.

The animals were housed in polypropylene cages with a stainless-steel top grill, with facilities for providing food and water, and paddy husk as the bedding. Regulated conditions were maintained, with a temperature of 23°C ± 4°C, humidity 30-70% and a 12-hour light-dark cycle. The animals were given free access to filtered water and commercial animal feed in the form of pellets. Forty-two Wistar rats of either gender, aged 2-3 months and weighing 200-250 g, were randomly selected and divided into the control and treatment groups.

For induction of OA, the rats were anaesthetised with ketamine (40-100 mg/kg) plus xylazine (5-13 mg/kg) [12], and osteoarthritis was induced by giving a single intra-articular injection of 2 mg MIA in 50 µl of saline solution via a 26.5-G needle in the left knee (hindlimb) [13].

**Figure 1:**
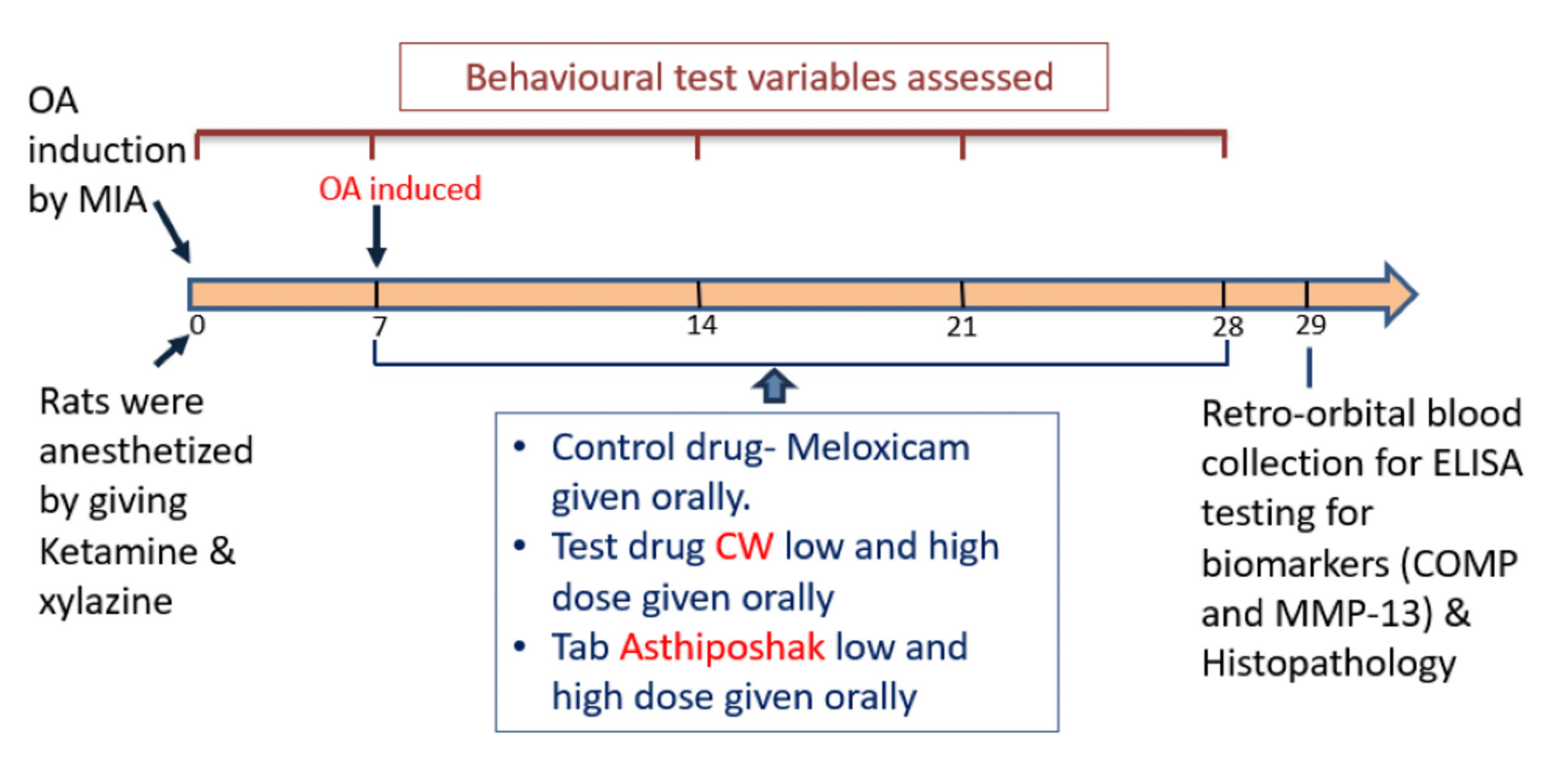
Brief overview of study procedure.

Table 1 provides details of the study groups, and Figure 1 illustrates the procedure.

**Table 1:** Study groups and dosing Dose calculation for *Commiphora wightii* and Asthiposhak was derived from references [14,15] and the following calculations. *C. wightii* human therapeutic dose is 500 mg thrice a day (1500 mg/day), animal dose is 135 mg/kg/day. Asthiposhak tablets human therapeutic dose is two tablets thrice a day (4500 mg/day) (Average weight per tablet is 750 mg), animal dose is 405 mg/kg/day. CW low dose (i.e., 90 mg/kg) corresponds to a human therapeutic dose of 1000 mg/day. CW high dose (i.e., 135 mg/kg) corresponds to a human therapeutic dose of 1500 mg/day. Asthiposhak low dose (i.e., 270 mg/kg) corresponds to a human therapeutic dose of 3000 mg/day (4 tablets/day). Asthiposhak high dose (i.e., 405 mg/kg) corresponds to a human therapeutic dose of 4500 mg/day (6 tablets/day). The study drugs (*C. wightii* (CW) and Asthiposhak (ASTP)) were provided by Shree Dhootapapeshwar Ltd; MIA and meloxicam were provided by Sigma Aldrich.

Group no.	Group name [n = 42 (n = 6/ group)]	Inducing agent	Drug and dose
1	Normal control (NC)	50 µl of saline via 26.5-G needle in left knee (day 0)	Oral 1 ml of 0.9% saline
2	Disease control (DC)	2 mg of MIA in 50 µl of saline intra-articularly via 26.5-G needle in left knee (day 0)	Oral 1 ml of 0.9% saline
3	Positive control (PC)		Oral meloxicam 1 mg/kg [9]
4	*Commiphora wightii* low dose (CW-LD)		Oral CW 90 mg/kg
5	*Commiphora wightii* high dose (CW-HD)		Oral CW 135 mg/kg
6	Asthiposhak low dose (ASTP-LD)		Oral ASTP 270 mg/kg
7	Asthiposhak high dose (ASTP-HD)		Oral ASTP 405 mg/kg

For process of induction of OA, on day 0 the rats were anaesthetised with ketamine (40-100 mg/kg) plus xylazine (5-13 mg/kg) [12], and by giving a single intra-articular injection of 2 mg MIA in 50 µl of saline solution via a 26.5-G needle in the left knee (hindlimb) OA was induced [13], behavioural test variables were assessed every 7 days on days 0, 7, 14, 21 and 28. Study drugs were administered orally from day 7 to 28. On day 29, retro-orbital blood was collected for enzyme-linked immunosorbent assay (ELISA) testing for biomarkers. After sacrificing the rats, the left knee joints were removed and sent for histopathology. Variables assessed were behavioural activity, histopathology, and biomarkers by ELISA.

Behavioural activity

The behavioural tests conducted were the rotarod test, the hot plate analgesiometer test, the grip strength test, and the open field test. The variables of disease activity were assessed on days 0 (baseline), 7, 14, 21, and 28.

Rotarod Test

The animals were placed on the rotarod at a speed of 4-5 rpm. The variable measured was the latency to falling off or the time spent on the rotarod in one minute. The animals were screened for their eligibility for this test before beginning the assessment [16]. The rats that could remain on the revolving drum for a minimum of 150 seconds were selected for drug testing.

Hot Plate Analgesiometer Test

The animals were placed on a hot plate analgesiometer at a temperature of 55°C with a cut-off period of 15 seconds. The variable assessed was time to lick the left hindlimb paw [16].

Grip Strength Test

To measure the grip strength, the animals were held with their forelimbs and left hindlimbs. The animals were then induced to grasp rigid bars attached to a grip strength metre with their left hindlimb. Each animal was gently pulled backwards, and the tension reading of the grip strength metre was recorded in newtons [13].

Open Field Test

All the animals were subjected to the open field test with video tracking (Maze Master software version 2) for assessment of locomotor activity (mobility). The variable assessed was the number of squares crossed in 5 minutes [17].

Histopathology analysis

Specimens from the centre of the knee joint were collected and fixed in 10% buffered neutral formalin for up to 5 days. All fixed specimens were washed in slowly running tap water for a minimum of 30 minutes. Excess soft tissue was stripped away from the knee joint to allow for greater surface area. After the excision of soft tissue, specimens were decalcified in 5% nitric acid in distilled water. Once the decalcification was completed, specimens were rinsed in water briefly and transferred to ammonia solution for 30 minutes to neutralise any acids left. The samples were then washed in running tap water thoroughly for up to 24 hours. After decalcification was deemed to be complete, the specimens were processed overnight in a tissue processor prior to embedding in molten paraffin wax at 60°C.

An intact flexed joint was embedded in paraffin. Sections were cut at 5 µm with a rotary microtome. Paraffin ribbons were attuned in a water bath at 40°C and collected onto microscope slides. Morphological tissue structure preservation was evaluated by haematoxylin and eosin (H&E) staining. The slides were assessed and scored by an independent veterinary pathologist, who graded the severity of arthritis as 0 = no degeneration, 1 = mild changes, 2 = moderate changes, 3= marked changes, and 4 = severe changes, based on the following parameters [18,19]: 1. Periarticular inflammatory cell infiltration; 2. Synovial inflammation; 3. Synovial membrane derangement (derangement, hyperplasia, and pannus formation); 4. Cartilage plate changes (derangement, degeneration, necrosis, erosion, and chondrocyte damage); 5. Bone changes

The histologic grading had a minimum of 0 and a maximum of 3.

Biomarker variables

On day 28, 2 ml of blood was collected through retro-orbital puncture in anaesthetised rats. The blood sample was centrifuged at 3000 rpm, and the serum was collected, divided equally, and stored in two aliquots at −80°C before performing ELISA for cartilage oligomeric matrix protein (COMP) and matrix metalloproteinase-13 (MMP-13) levels. Kinesis Dx ELISA kits purchased from Krishgen Biosystems, Mumbai, Maharashtra-400018, were used for the assay.

Statistical analysis

Results were expressed as mean ± SD. GraphPad Instat software version 3.10 was used for statistical analysis. Normality of data was assessed using the Kolmogorov-Smirnov test. Analysis of variance (ANOVA) with post hoc Tukey’s test was used to compare different variables among groups for parametric data. Non-parametric data were analysed using the Kruskal-Wallis test with post hoc Dunn’s test.

## Results

Behavioural activity variable results

Rotarod Test

**Figure 2:**
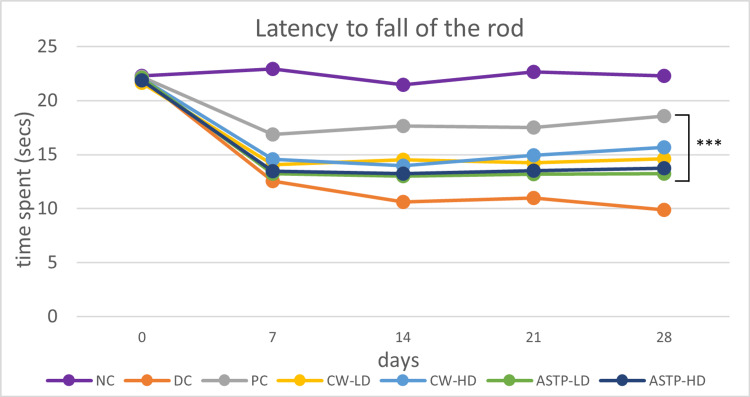
Results of the rotarod test ***P < 0.001 vs DC using one-way ANOVA followed by post hoc Tukey’s test NC: Normal control; DC: Disease control; PC: Positive control; CW-LD: *Commiphora wightii* low dose; CW-HD: *Commiphora wightii* high dose; ASTP-LD: Asthiposhak low dose; ASTP-HD: Asthiposhak high dose

After 28 days of treatment, all treatment groups showed a significant (p < 0.001) increase in time spent on the rotarod (positive control (PC), *C. wightii* low dose (CW-LD), *C. wightii* high dose (CW-HD), ASTP-LD, and ASTP-HD groups) compared to the disease control (DC) group. The increase was more significant for the CW-HD group than for the ASTP-LD and ASTP-HD groups (Figure 2).

Hot Plate Analgesiometer Test

**Figure 3:**
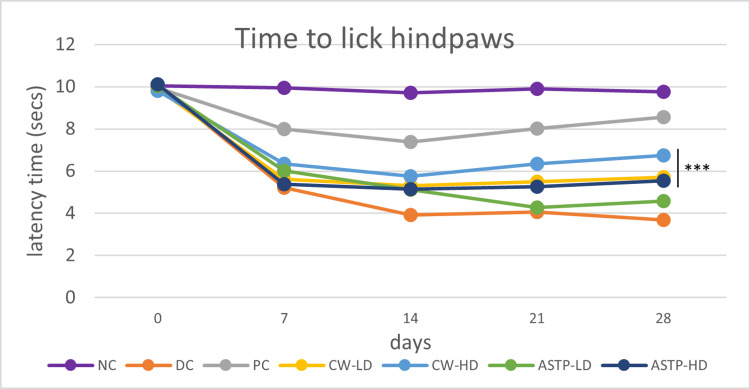
Results of the hot plate analgesiometer test ***P < 0.001 vs DC using one-way ANOVA followed by post hoc Tukey’s test NC: Normal control; DC: Disease control; PC: Positive control; CW-LD: *Commiphora wightii* low dose; CW-HD: *Commiphora wightii* high dose; ASTP-LD: Asthiposhak low dose; ASTP-HD: Asthiposhak high dose

After 28 days of treatment, the pain threshold of the CW-LD, CW-HD, and ASTP-HD groups was significantly (p < 0.001, p < 0.001, and p < 0.01) higher than that of the ASTP-LD group and DC group. The pain threshold of the CW-HD group was significantly (p < 0.001) higher than that of the CW-LD and ASTP-HD groups (Figure 3).

Grip Strength

**Figure 4:**
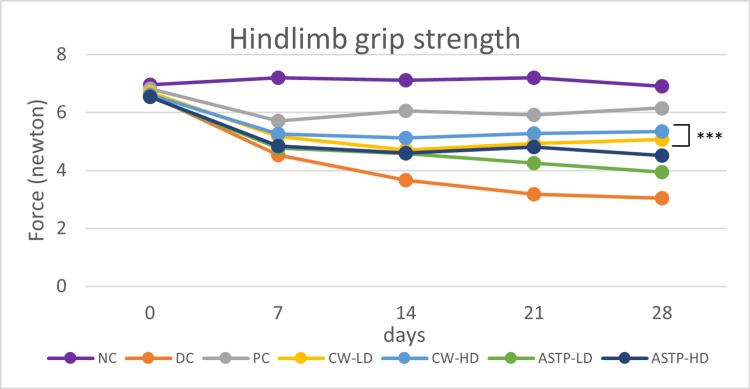
Results of the grip strength test ***P < 0.001 vs DC using one-way ANOVA followed by post hoc Tukey’s test NC: Normal control; DC: Disease control; PC: Positive control; CW-LD: *Commiphora wightii* low dose; CW-HD: *Commiphora wightii* high dose; ASTP-LD: Asthiposhak low dose; ASTP-HD: Asthiposhak high dose

After 28 days of treatment, all treatment groups showed a significant (p < 0.001) increase in grip strength (PC, CW-LD, CW-HD, ASTP-LD and ASTP-HD groups) compared to the DC group. The grip strength in the CW-LD and CW-HD groups was significantly (p < 0.001) higher than in the ASTP-LD group and DC group (Figure 4).

Open Field Test

**Figure 5:**
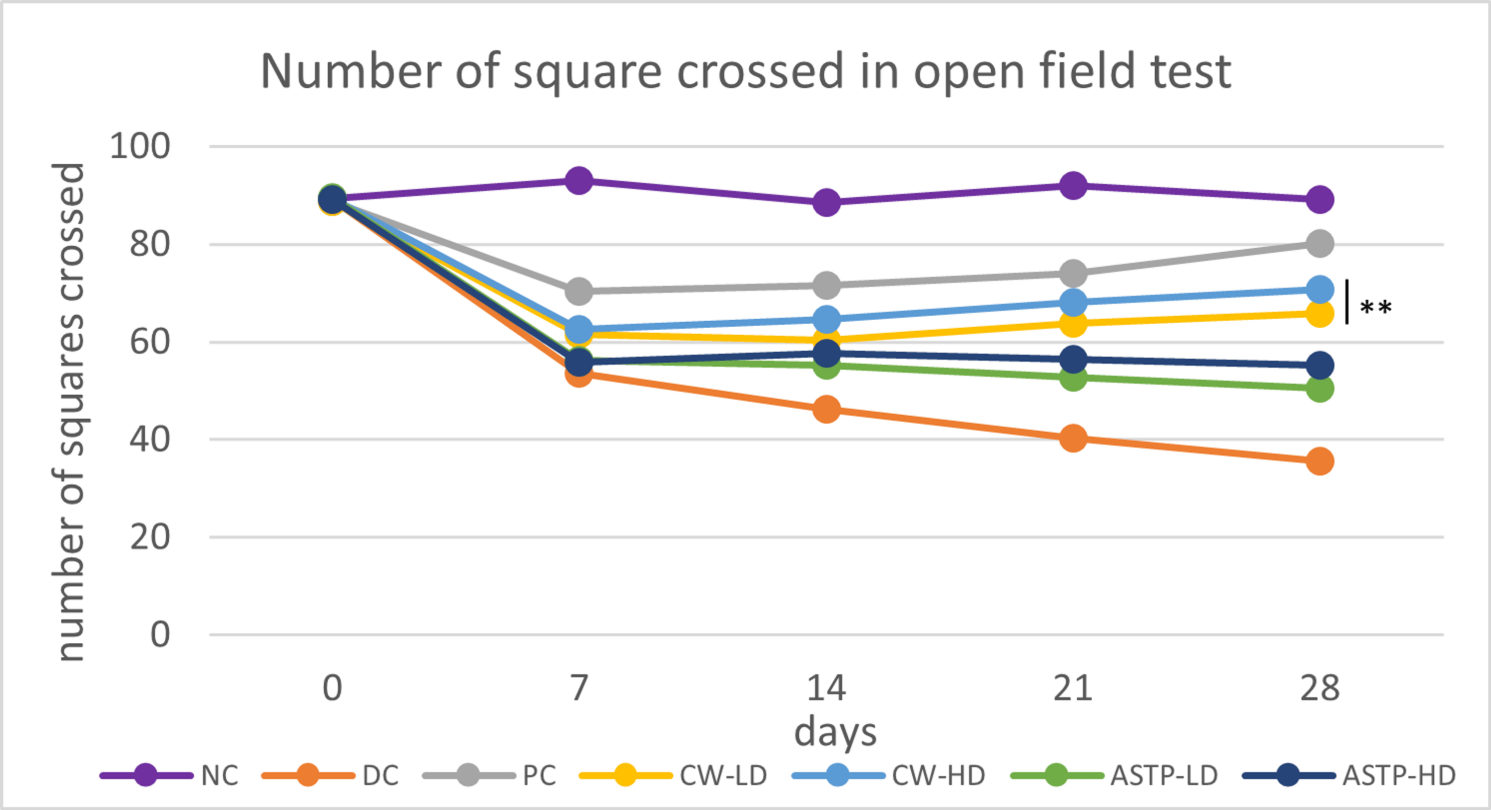
Results of the open field test ***P < 0.001 vs DC using one-way ANOVA followed by post hoc Tukey’s test NC: Normal control; DC: Disease control; PC: Positive control; CW-LD: *Commiphora wightii* low dose; CW-HD: *Commiphora wightii* high dose; ASTP-LD: Asthiposhak low dose; ASTP-HD: Asthiposhak high dose

After 28 days of treatment, the CW-LD, CW-HD, ASTP-LD and ASTP-HD groups were comparable with the PC group in the open field test. The number of squares crossed was significantly higher (p < 0.01) in the CW-LD group than in the ASTP-LD group. Moreover, the number of squares crossed was significantly higher (p < 0.001) in the CW-HD group than in the ASTP-LD and ASTP-HD groups and the DC group (Figure 5).

Biomarker analysis results

**Figure 6:**
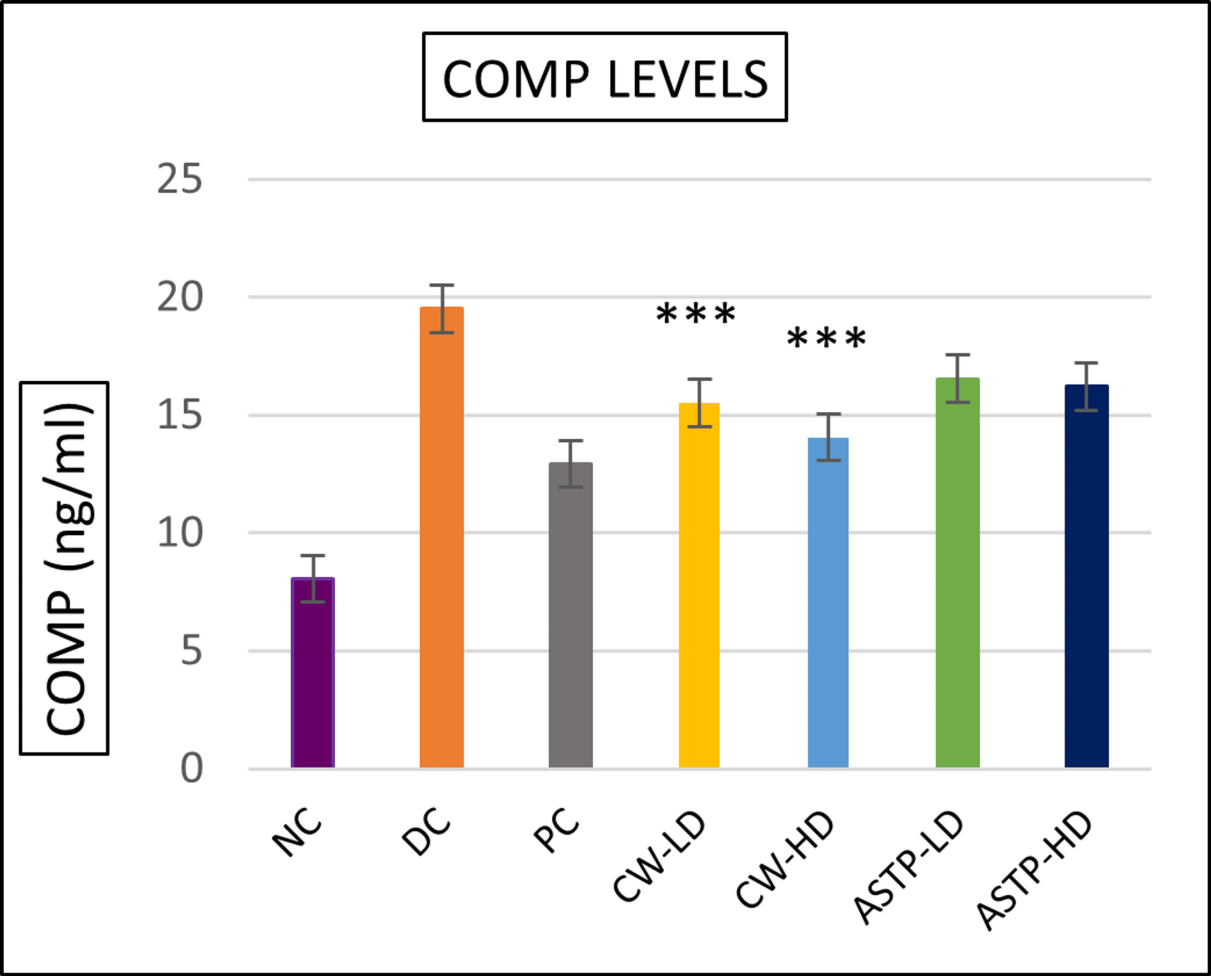
COMP biomarker levels *** P < 0.001 vs DC using one-way ANOVA followed by post hoc Tukey’s test NC: Normal control; DC: Disease control; PC: Positive control; CW-LD: *Commiphora wightii* low dose; CW-HD: *Commiphora wightii* high dose; ASTP-LD: Asthiposhak low dose; ASTP-HD: Asthiposhak high dose; COMP: cartilage oligomeric matrix protein

**Figure 7:**
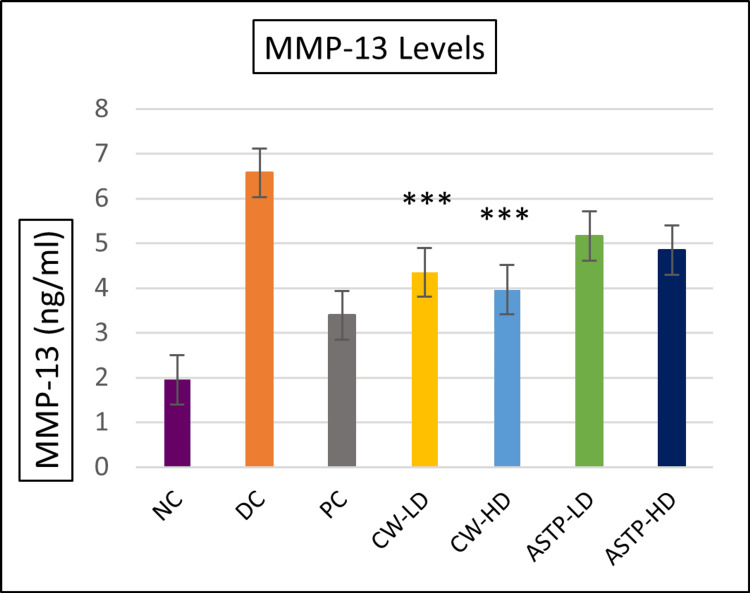
MMP-13 biomarker levels ***P < 0.001 vs DC using one-way ANOVA followed by post hoc Tukey’s test NC: Normal control; DC: Disease control; PC: Positive control; CW-LD: *Commiphora wightii* low dose; CW-HD: *Commiphora wightii* high dose; ASTP-LD: Asthiposhak low dose; ASTP-HD: Asthiposhak high dose; MMP-13: matrix metalloproteinase-13

As shown in Figures 6-7, COMP and MMP-13 levels were significantly lower in CW both doses than in the DC group. The COMP and MMP-13 ELISA test showed that CW-LD and CW-HD were highly significant (p < 0.001) compared to the DC group.

Histopathology of the left knee joint results

**Figure 8:**
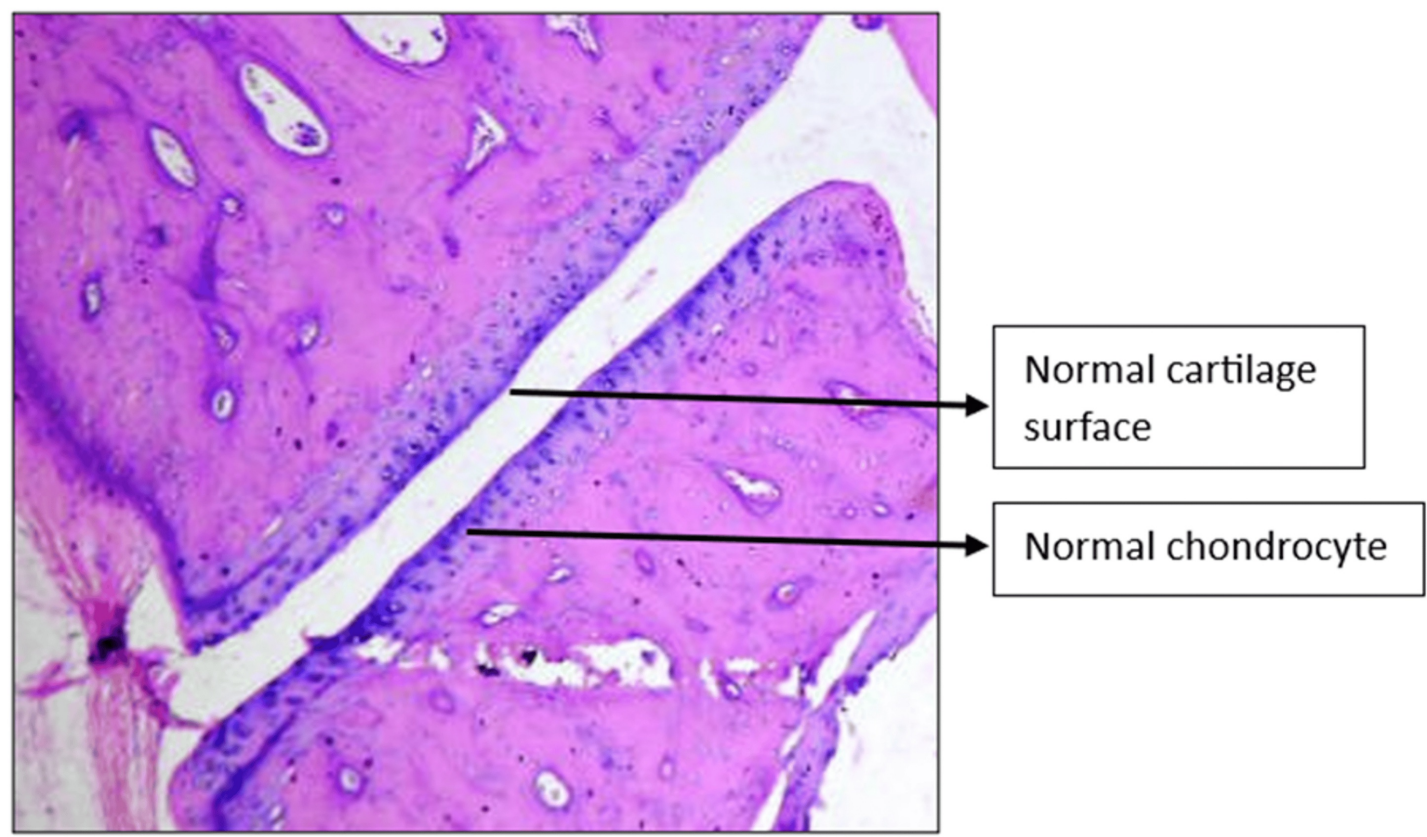
H&E stain of the left knee joint (100×) in the normal control group

**Figure 9:**
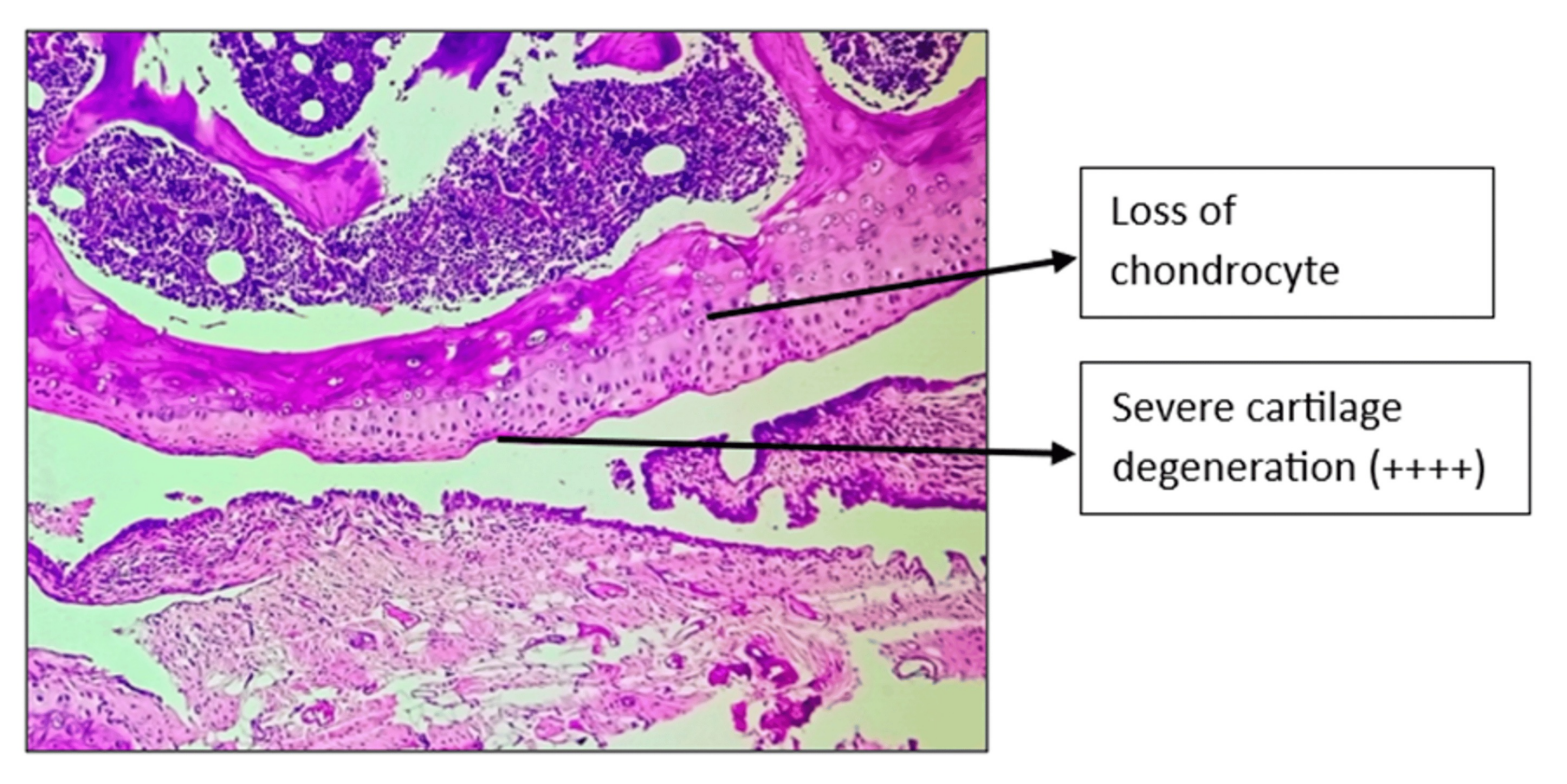
H&E stain of left knee joint (100×) in disease control group

**Figure 10:**
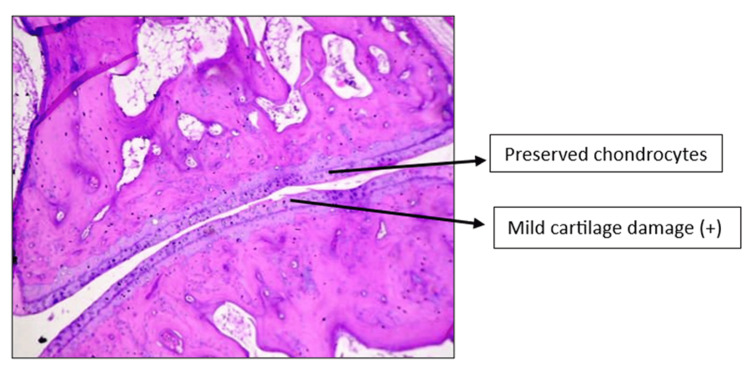
H&E stain of left knee joint (100×) in positive control group

**Figure 11:**
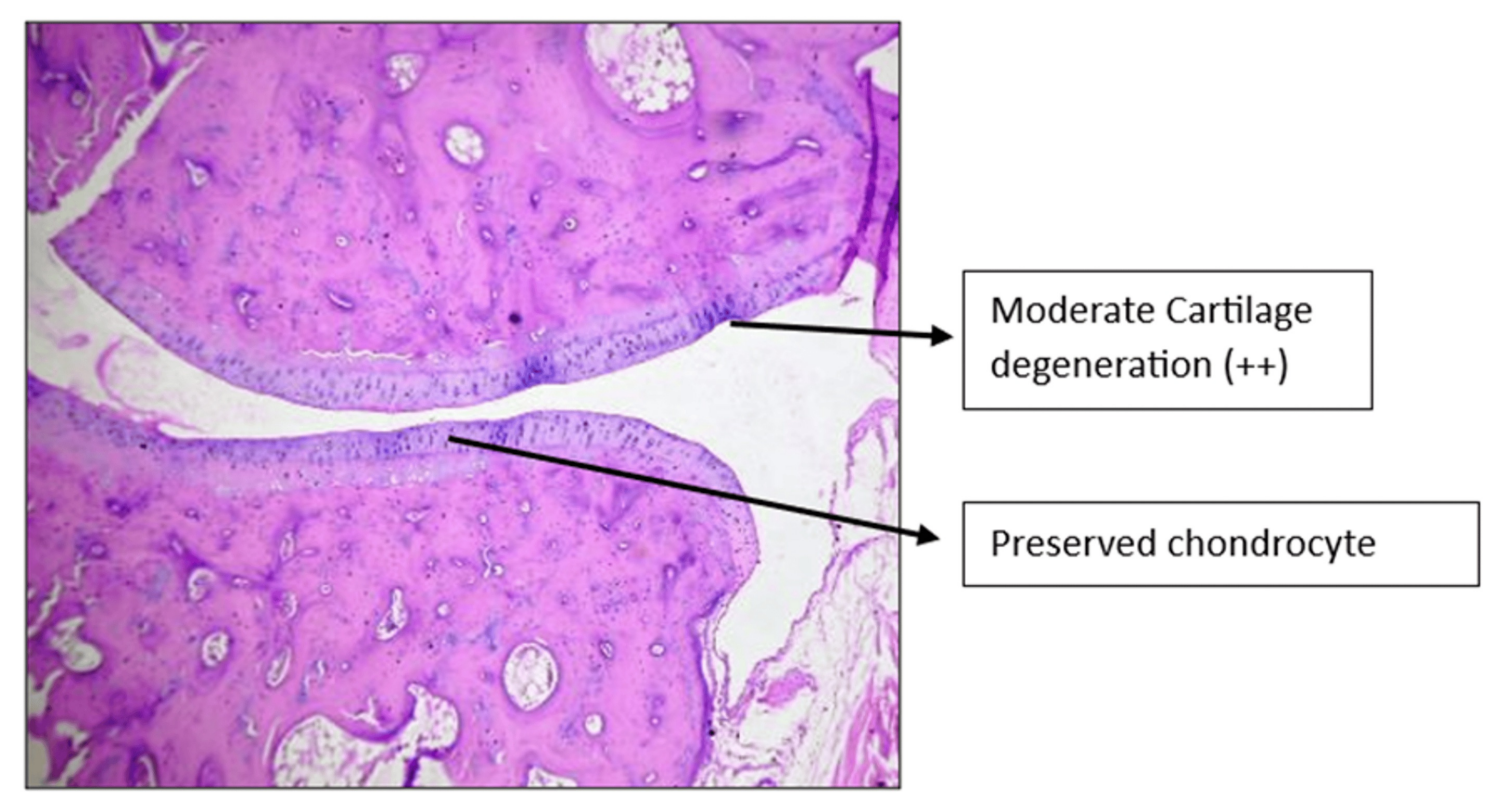
H&E stain of left knee joint (100×) in Commiphora wightii low-dose group

**Figure 12:**
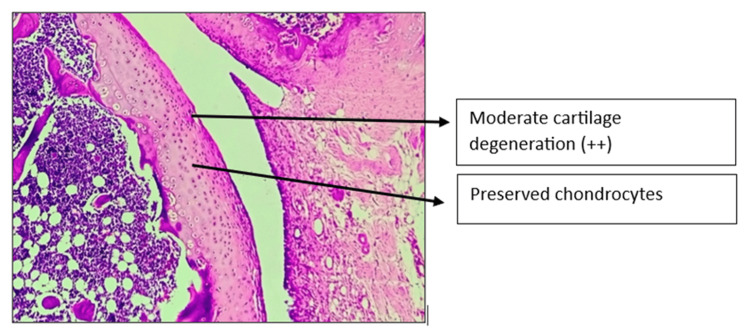
H&E stain of left knee joint (100×) in Commiphora wightii high-dose group

**Figure 13:**
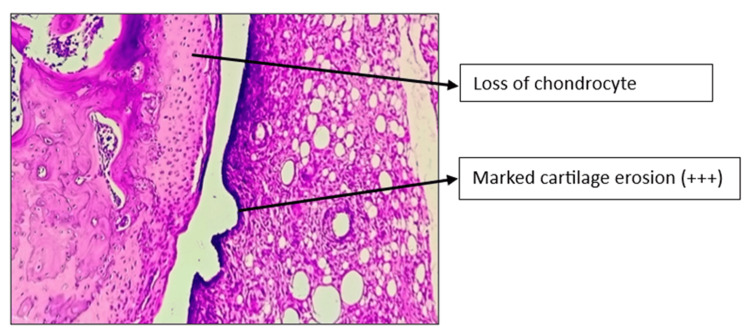
H&E stain of left knee joint (100×) in Asthiposhak low-dose group

**Figure 14:**
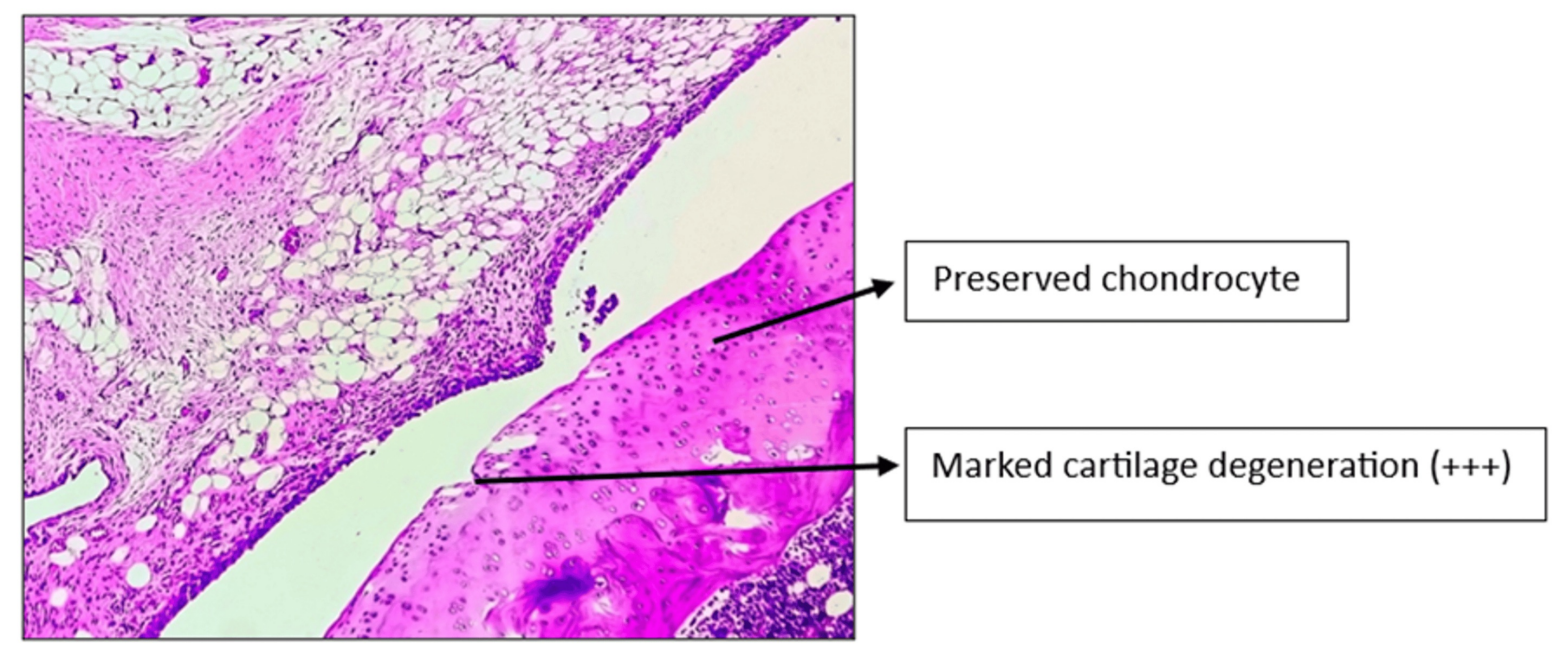
H&E stain of left knee joint (100×) in Asthiposhak high-dose group

The CW-LD and CW-HD groups exhibited a significant reduction (p < 0.01) in the grade of arthritis compared to the DC group. Histopathologic grading of the CW-LD group (median 2, interquartile range (IQR) 2-2), CW-HD group (median 1.5, IQR 1-2) and positive control group (median 1, IQR 0.25-1) shows a significant reduction compared to the DC group (median 3, IQR 3-3). Treatment groups were compared with DC using the Kruskal-Wallis test followed by post hoc Dunn’s test (Figures 8-14).

## Discussion

In the behavioural test, histopathology and biomarker study, this study found that CW and ASTP showed anti-osteoarthritic and chondroprotective effects.

In a 2014 study by Manjhi et al., the CW or Commiphora Mukul or Balsamodendron Mukul extract (40%) groups after 30 days of treatment stayed on the rotarod close to baseline levels of day 5, suggesting an improvement in motor coordination by reduction in pain [20]. In the hot plate test, treatment with Balsamodendron Mukul extract (40%) resulted in a decrease in paw withdrawal latency, indicating a reduction in pain perception and relief of pain. The oral dose of gum resin extract has been shown to relieve OA pain, regenerate the cartilaginous matrix and increase subchondral bone components, which is similar to the results of the present study, where, after treatment, on day 28, both CW doses and both ASTP doses showed significant improvement and increase in the time spent on the rotarod. In the hot plate analgesimeter test, both CW doses and ASTP-HD showed significant reduction in pain perception and relief of pain. These findings indicate a positive role of CW and Asthiposhak tablets in improving motor balance coordination and reducing pain.

The study found that in both CW doses and ASTP-HD, the number of squares crossed increased from baseline after day 28 of treatment, as compared to the DC group, indicating improvement in the mobility of the rats. No studies have evaluated the open field test for CW or Asthiposhak individually in an OA model. This is the only study that demonstrates the effect of CW and Asthiposhak in an open field test. Moreover, in the grip strength test, the left hindlimb paw grip strength significantly increased in the CW-HD, ASTP-LD and ASTP-HD groups compared to the DC group. No studies have evaluated the grip strength test for CW and Asthiposhak in an OA model. This is the only study showing the effect of CW and Asthiposhak on grip strength. Although there have been no previous studies of Asthiposhak in OA, it was studied for osteoporosis by Sanaye et al. in 2021 [15]. Femur bone histopathology revealed increased trabecular thickness and decreased osteoclast formation, and the calcium content in bone ash was significantly increased after Asthiposhak treatment, indicating remineralisation of bones. Bone mineral density was also significantly reversed in Asthiposhak-treated animals. Similar to osteoporosis, OA is common in the elderly population. Hence, this study was planned to examine the effect of Asthiposhak on Osteoarthritis.

Biomarker analysis has previously not been performed in CW- or Asthiposhak-treated animals. The present study was intended to confirm the chondroprotective effect of both study drugs in low and high doses by analysing COMP and MMP-13, which are the biomarkers used to assess cartilage health [21,22]. The study evaluated CW and the CW-containing proprietary formulation Asthiposhak in two different doses to provide more substantial evidence. MMP-13 levels are upregulated in OA patients, which plays an important role in joint destruction by breaking down type II collagen [23]. Inhibiting MMP-13 has emerged as a new area of interest in the treatment of OA [24]. CW and Asthiposhak reduced the levels of MMP-13 and COMP. CW-HD and CW-LD were statistically significant compared to the DC group. Based on the combined results of the behavioural assessments, histopathology analysis and chondroprotective biomarkers (COMP and MMP-13), it can be inferred that although both doses of CW and of Asthiposhak possess promising chondroprotective potential, those of CW and the high dose of Asthiposhak had a more pronounced therapeutic effect.

This study has a few limitations. There was no sample size calculation. Furthermore, randomisation and blinding were not used for behavioural testing. The veterinary pathologist was blinded for histopathology grading, however. The study results are generalisable only to the same species, as the construct validity may differ between species. X-ray radiography and examination of other biomarkers of inflammation could also have been performed.

## Conclusions

This investigation showed that in the MIA-induced OA model in Wistar rats, both CW and Asthiposhak have significant anti-osteoarthritic and chondroprotective effects, especially at higher dosages compared to the DC group. The treated groups showed improved mobility, pain tolerance and motor coordination in behavioural tests, including the rotarod, hot plate, grip strength, and open field tests. These enhancements were on par with or better than those observed with the standard control medication, meloxicam. Moreover, CW and Asthiposhak considerably reduced the serum biomarkers COMP and MMP-13, which are known to be elevated in OA and linked to cartilage degradation and attenuated histopathological alterations in the knee joint. The high dose of Asthiposhak was particularly effective, confirming the idea of a dose-dependent therapeutic benefit, whereas CW, at both low and high doses, demonstrated the most consistent outcomes across all parameters.

This preclinical data supports the need to investigate CW and Asthiposhak as possible treatment agents for OA. More research is necessary, including clinical trials, to confirm their safety and effectiveness in humans and to explore their mechanism of action.

## References

[1] Englund M (2023). Osteoarthritis, part of life or a curable disease? A bird's-eye view. J Intern Med.

[2] Heidari B (2011). Knee osteoarthritis prevalence, risk factors, pathogenesis and features: Part I. Caspian J Intern Med.

[3] Cui A, Li H, Wang D, Zhong J, Chen Y, Lu H (2020). Global, regional prevalence, incidence and risk factors of knee osteoarthritis in population-based studies. EClinicalMedicine.

[4] Singh A, Das S, Chopra A (2022). Burden of osteoarthritis in India and its states, 1990-2019: Findings from the Global Burden of disease study 2019. Osteoarthritis Cartilage.

[5] Maldonado M, Nam J (2013). The role of changes in extracellular matrix of cartilage in the presence of inflammation on the pathology of osteoarthritis. Biomed Res Int.

[6] Aweid O, Haider Z, Saed A, Kalairajah Y (2018). Treatment modalities for hip and knee osteoarthritis: A systematic review of safety. J Orthop Surg (Hong Kong).

[7] Watt FE, Gulati M (2017). New drug treatments for osteoarthritis: What is on the horizon?. Eur Med J Rheumatol.

[8] Bendele AM (2001). Animal models of osteoarthritis. J Musculoskelet Neuronal Interact.

[9] Nagy E, Vajda E, Vari C, Sipka S, Fárr AM, Horváth E (2017). Meloxicam ameliorates the cartilage and subchondral bone deterioration in monoiodoacetate-induced rat osteoarthritis. PeerJ.

[10] Ahmad MA, Mujeeb M, Akhtar M, Khushtar M, Arif M, Haque MR (2020). Guggulipid: A promising multi-purpose herbal medicinal agent. Drug Res (Stuttg).

[11] Kumar K, Godatwar P, Sharma S (2023). A pilot, open-label, proof-of-concept study to evaluate the efficacy and safety of Asthiposhak® tablets in participants suffering from Asthikshaya or osteopenia. Cureus.

[12] (2025). Anesthesia (Guideline) | Vertebrate Animal Research. https://animal.research.uiowa.edu/iacuc-guidelines-anesthesia.

[13] Marker CL, Pomonis JD (2012). The monosodium iodoacetate model of osteoarthritis pain in the rat. Methods Mol Biol.

[14] Sarup P, Bala S, Kamboj S (2015). Pharmacology and phytochemistry of oleo-gum resin of Commiphora wightii (Guggulu). Scientifica (Cairo).

[15] Sanaye M, Bora B, Chawda M, Kshirsagar V (2021). Evaluation of anti-osteoporotic activity of Asthiposhak tablets in ovariectomized rats. Int J Pharm Sci Res.

[16] Piel MJ, Kroin JS, van Wijnen AJ, Kc R, Im HJ (2014). Pain assessment in animal models of osteoarthritis. Gene.

[17] Han J, Park D, Han S (2023). Animal Models in Osteoarthritis Research: Pain Behavioral Methods and Clinical Significance. Int J Pain.

[18] Gerwin N, Bendele AM, Glasson S, Carlson CS (2010). The OARSI histopathology initiative - recommendations for histological assessments of osteoarthritis in the rat. Osteoarthritis Cartilage.

[19] Kobayashi K, Imaizumi R, Sumichika H, Tanaka H, Goda M, Fukunari A, Komatsu H (2003). Sodium iodoacetate-induced experimental osteoarthritis and associated pain model in rats. J Vet Med Sci.

[20] Manjhi J, Rawat B, Sinha A (2014). Effect of Balsamodendron Mukul gum resin extract on pain response in osteoarthritic rats. Asian J Pharm Clin Res.

[21] Hoch JM, Mattacola CG, Medina McKeon JM, Howard JS, Lattermann C (2011). Serum cartilage oligomeric matrix protein (sCOMP) is elevated in patients with knee osteoarthritis: A systematic review and meta-analysis. Osteoarth Cartil.

[22] Attur M, Krasnokutsky-Samuels S, Samuels J, Abramson SB (2013). Prognostic biomarkers in osteoarthritis. Curr Opin Rheumatol.

[23] Burrage PS, Mix KS, Brinckerhoff CE (2006). Matrix metalloproteinases: Role in arthritis. Front Biosci.

[24] Li H, Wang D, Yuan Y, Min J (2017). New insights on the MMP-13 regulatory network in the pathogenesis of early osteoarthritis. Arthritis Res Ther.

